# Altered Motor Unit Discharge Coherence in Paretic Muscles of Stroke Survivors

**DOI:** 10.3389/fneur.2017.00202

**Published:** 2017-05-15

**Authors:** Chenyun Dai, Nina L. Suresh, Aneesha K. Suresh, William Zev Rymer, Xiaogang Hu

**Affiliations:** ^1^Joint Department of Biomedical Engineering, University of North Carolina at Chapel Hill and North Carolina State University, Raleigh, NC, USA; ^2^Sensory Motor Performance Program, Rehabilitation Institute of Chicago, Chicago, IL, USA; ^3^Department of Physical Medicine and Rehabilitation, Feinberg School of Medicine, Northwestern University, Chicago, IL, USA; ^4^Committee on Computational Neuroscience, University of Chicago, Chicago, IL, USA

**Keywords:** motor unit, coherence, stroke, synchronization, surface electromyogram

## Abstract

After a cerebral stroke, a series of changes at the supraspinal and spinal nervous system can alter the control of muscle activation, leading to persistent motor impairment. However, the relative contribution of these different levels of the nervous system to impaired muscle activation is not well understood. The coherence of motor unit (MU) spike trains is considered to partly reflect activities of higher level control, with different frequency band representing different levels of control. Accordingly, the objective of this study was to quantify the different sources of contribution to altered muscle activation. We examined the coherence of MU spike trains decomposed from surface electromyogram (sEMG) of the first dorsal interosseous muscle on both paretic and contralateral sides of 14 hemispheric stroke survivors. sEMG was obtained over a range of force contraction levels at 40, 50, and 60% of maximum voluntary contraction. Our results showed that MU coherence increased significantly in delta (1–4 Hz), alpha (8–12 Hz), and beta (15–30 Hz) bands on the affected side compared with the contralateral side, but was maintained at the same level in the gamma (30–60 Hz) band. In addition, no significant alteration was observed across medium–high force levels (40–60%). These results indicated that the common synaptic input to motor neurons increased on the paretic side, and the increased common input can originate from changes at multiple levels, including spinal and supraspinal levels following a stroke. All these changes can contribute to impaired activation of affected muscles in stroke survivors. Our findings also provide evidence regarding the different origins of impaired muscle activation poststroke.

## Introduction

After a cerebral stroke, a series of changes at the spinal and supraspinal levels of the nervous system can influence the control of muscle activation, leading to different motor impairment. One convenient way of identifying the different levels of contributions to altered muscle activation is to characterize the discharge patterns of motor unit (MU), given that MU discharge activities at the populational level can now be readily obtained from the skin surface (1, 2). Since different alpha motor neurons receive common synaptic excitations from spinal and supraspinal pathways during sustained contraction (3–6), the discharging times of MU should exhibit some degree of correlation. The cross-correlation analysis of MU spike trains in the time domain has been widely used to study the connectivity between the motor neuron pool and the spinal or cortical inputs (7–11). However, the relative strength of correlation across different levels cannot be systematically quantified using this approach.

In contrast, the coherence analysis, which reflects the cross-correlation of MU spike trains in the frequency domain, provides complementary and important information from another perspective that can reveal these activities of higher level control (12–14). Previous studies have established the findings that the coherence of MU spike trains under 60 Hz is critically important and can be separated into four different frequency bands, including delta band (1–4 Hz), alpha band (8–12 Hz), beta band (15–30 Hz), and gamma band (30–60 Hz) (5, 15–17). Moreover, physiological origins for the synchronization of each band have been widely accepted. Specifically, the delta band is thought to reflect the common modulation of firing rates (18, 19); the alpha band highly depends on the feedback from muscle spindles and possibly results from rhythmical activities of the spinal reflex loop (20, 21); the beta band may reflect cortical and subcortical activities (15, 19); and the gamma band also represents cortical activities (19).

Previous studies have found that the MU synchronization of certain frequency bandwidths change under atypical conditions. A recent study reported an increase of MU coherence in delta, alpha, and beta bands after muscle fatigue (17). Another recent study (22) examined the variation of coherence with muscle pain. Their results indicated that muscle pain led to an increase in the coherence of MU spike trains in the delta band, but a decrease in the alpha band. Given the direct and indirect cortical and subcortical projections to the motor neuron pool, it can be conjectured that the alteration of synaptic inputs to motor neurons may influence the MU coherence at different frequency bandwidths. To date, few studies have examined the possible changes of MU synchronization in stroke survivors (23, 24). These studies were mostly based on electroencephalogram—electromyogram (EMG) synchronization rather than the coherence of MU spike train itself—and did not specifically quantify the variation of coherence in each frequency corresponding to specific physiological implications.

Accordingly, the objective of our current study was to systematically investigate the potentially altered control at different levels in contribution to impaired finger muscle activation in stroke survivors. In our study, MU spike trains were acquired from the decomposition of surface electromyogram (sEMG) of the first dorsal interosseous (FDI) muscle at a wide range of index finger abduction forces on both paretic and contralateral sides of hemispheric stroke survivors. The discharge coherence of concurrently active MUs was calculated across four frequency bandwidths at different force levels and was compared between the affected and contralateral muscles. The finger muscle was examined because stroke survivors tend to show persistent impairment in finger muscle activation. Particularly, the FDI muscle was tested because FDI is the only muscle involved in index finger abduction force, which can avoid force contribution of other muscles, and because the FDI is superficial and is accessible from the skin surface during EMG recordings. Our findings provide evidence that there are substantial changes in the common input, arising from the spinal and supraspinal circuitry, to the motor neuron pool innervating affected muscles, which can modify the control of muscle activation of stroke survivors.

## Materials and Methods

### Experimental Apparatus and Procedures

#### Participants

Experimental data from 14 chronic hemiparetic stroke participants (detailed demographic information is shown in Table 1) were acquired at the Rehabilitation Institute of Chicago. All participants provided written informed consent. The experimental protocols were approved by the Institutional Review Board (#STU00084379) at Northwestern University.

**Table 1:** **Participant demographic information**.

Participant ID	Gender	Age	Years	Side	Chedoke	Fugl-Meyer
1	M	61	4	R	6	63
2	F	62	15	L	2	17
3	F	59	23	R	2	22
4	M	66	9	L	4	16
5	F	53	3	R	6	63
6	F	58	5	R	4	38
7	F	71	7	R	6	66
8	M	60	11	R	5	52
9	M	61	7	R	4	3
10	F	69	15	L	4	30
11	M	58	4	R	5	45
12	F	59	1	L	3	20
13	F	62	9	R	6	53
14	M	48	7	L	5	60

*Age, year of age; Years, years since stoke; Side, paretic side; Chedoke, Chedoke-McMaster stroke assessment ranging from 1 to 7, with 1 being the most severe impairment; Fugl-Meyer, Fugl-Meyer assessment ranging from 1 to 66, with 1 being the most severe impairment; M, male; F, female; R, right; L, left*.

The major inclusion criteria for stroke participants included (1) ability to provide written informed consent; (2) ability to communicate with and understand the instructions of the experimenter; (3) the duration since stroke >6 months; (4) impairment level of hand function measured with the Chedoke-McMaster score ranged from 2 to 6; (5) no medication; (6) no upper extremity inflammation, recent injury, pain, or other concurrent severe medical illness; (7) no history of vascular impairment.

#### Experimental Setup

**Figure 1:**
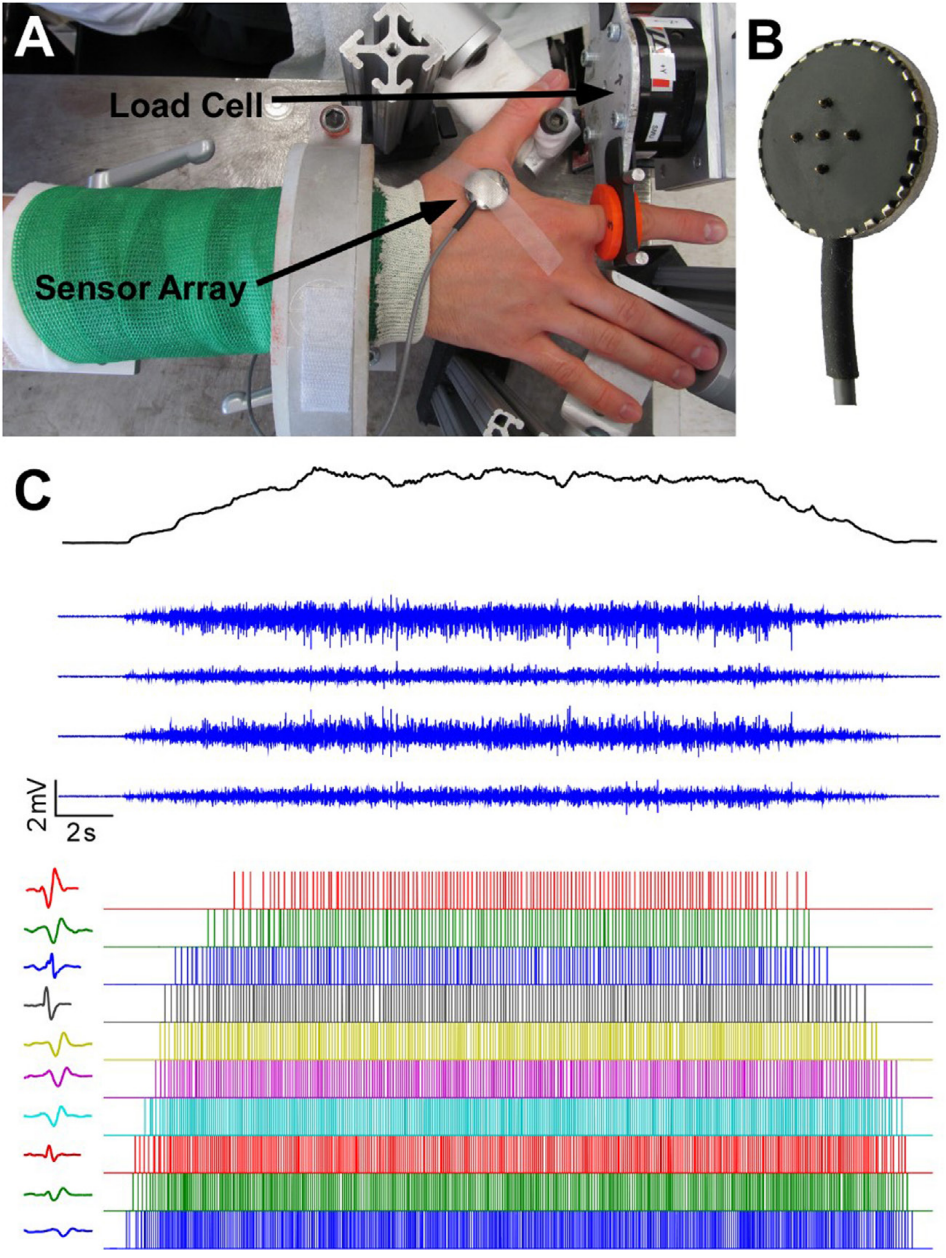
**Experimental apparatus, electromyogram recordings and motor unit decomposition**. **(A)** Experimental setup. **(B)** The five-probe sensor array. **(C)** The four-channel surface electromyogram (sEMG) signals during a trapezoidal force production. In addition, motor unit spike trains and corresponding templates after sEMG decomposition are also displayed.

The detailed information about the experimental setup has been reported in a previous study (25, 26). Briefly, participants sat in the experimental apparatus with their upper arm comfortably placed on a support, their forearm oriented at a full pronation position, secured with a cast and located in a ring mount interface attached to a forearm rest, and their wrist held neutral with respect to flexion/extension (Figure 1A). Their middle, ring, and little fingers were abducted away from the index finger resting on a supporting frame. The index finger casted and fixed to another ring mount interface was placed at approximately 60° apart from the thumb and directly attached to a 6 degree-of-freedom load cell (ATI, Inc., Apex, NC, USA). The recorded isometric index finger abduction forces were low-pass filtered at 200 Hz cutoff frequency and sampled at 2 kHz, and a high sampling rate is required to avoid aliasing effect from channel cross-talk. The participant’s skin above the FDI muscle was scrubbed with alcohol pads to reduce background noise. A five-pin sensor array (Delsys, Inc., Natick, MA, USA) was placed on the skin surface. Five 0.5-mm diameter cylindrical probes are located at the center of sensor array within a 5 mm × 5 mm square (Figure 1B). The sensor array and a reference electrode were connected to a Delsys Bagnoli sEMG system to record sEMG signals generated by the pairwise differentiation of these five electrodes. The sEMG recordings were amplified by a 1,000 gain and filtered with a bandwidth of 20–2 kHz with a sampling frequency at 20 kHz (Figure 1C).

#### Procedures

The data collection included two main sessions (one for each side) with same experimental protocols. Before the two main sessions, participants were required to perform maximum voluntary contractions (MVCs) for 3 s. Three repeated trials with 60 s rest between trials were tested to obtain the largest force value, which was designated as the MVC. To ensure a fair comparison between two sides, the MVC of paretic side was used during the force tasks on the contralateral side, such that the two sides produced the same absolute forces. Then, participants performed five repeated trials for five isometric contraction levels at 20, 30, 40, 50, and 60% MVC. The force trajectory contained a 5-s quiescent period for baseline noise calculation, an up-ramp increasing at 10% MVC/s, a constant force at prescribed MVC for 12 s, a down-ramp decreasing at 10% MVC/s, and a final 3-second quiescent period. The order of the force levels was randomly selected for each participant. One minute of rest period was provided between trials to avoid cumulative fatigue.

### Data Analysis

#### Preprocessing

The sEMG and force recordings selected for further data analysis must satisfy the following requirements: (1) no sudden change in the up-ramp force; (2) the variability of force during steady state within ±2 SDs of background force level; (3) the sEMG signal with the baseline noise level within ±10 μV, and signal-to-noise ratio greater than 5 (signal-to-noise ratio was defined as peak–peak amplitude of the EMG signal at steady state contractions divided by peak–peak amplitude of the baseline noise). Based on the criteria above, three trials for each MVC level per participant were finally selected for the coherence analysis. During the analysis, the accepted number of MU must be over eight to be included in the analysis because the large number of MUs resulted in an accurate coherence estimate.

#### MU Acceptance

All raw sEMG recordings were automatically decomposed using Nawab’s algorithm (1). The autodecomposition algorithm extracted the firing times, and four different MU action potential waveforms (from the four-channel EMG recordings) of each identified MU spike train. Then, a robust postexamination method, spike trigger average (STA) algorithm (27), was used to determine which MUs were retained for further analysis. The STA method reestimated MU action potential templates based on the firing times, MU action potential templates, and raw EMG signals obtained from Delsys. A high agreement of two different algorithms for each MU provided confidence regarding the reliability of decomposition results. The STA method calculated the coefficient of variation (CV) for peak–peak amplitude of MU action potential templates and the maximum correlation coefficient between STA MU action potential estimation and Delsys MU action potential templates. Only MUs with a mean correlation >0.7 and CV <0.3 across four channels were selected for data analysis based on our previous studies (17, 27, 28).

#### Coherence Calculation

**Figure 2:**
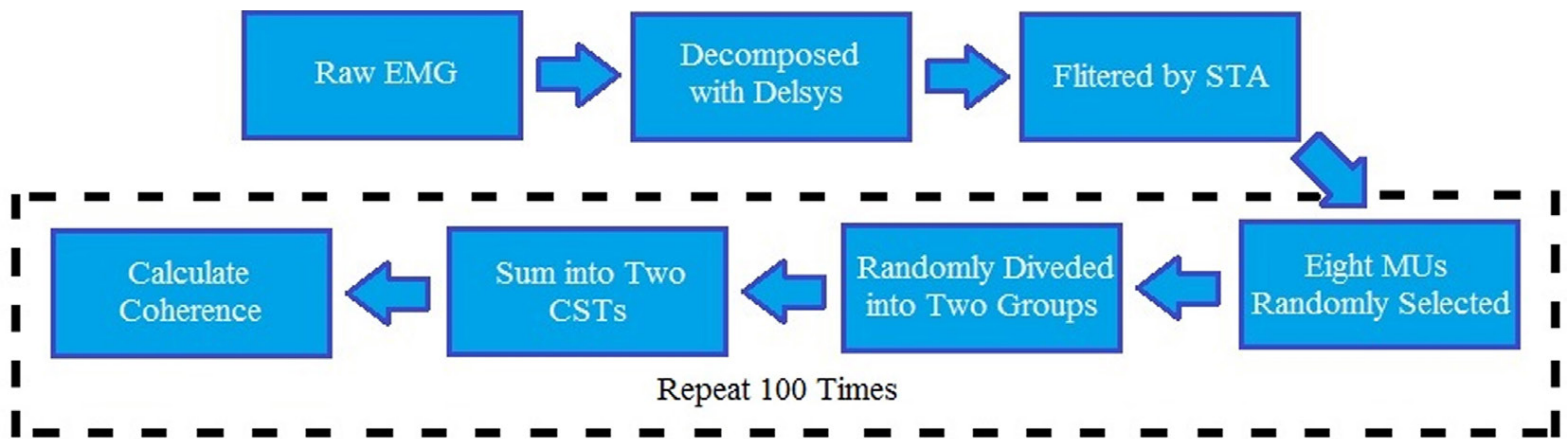
**Block diagram for the entire data analysis**. EMG, electromyography; STA, spike trigger average algorithm; CST, composite spike train; MU, motor unit.

**Figure 3:**
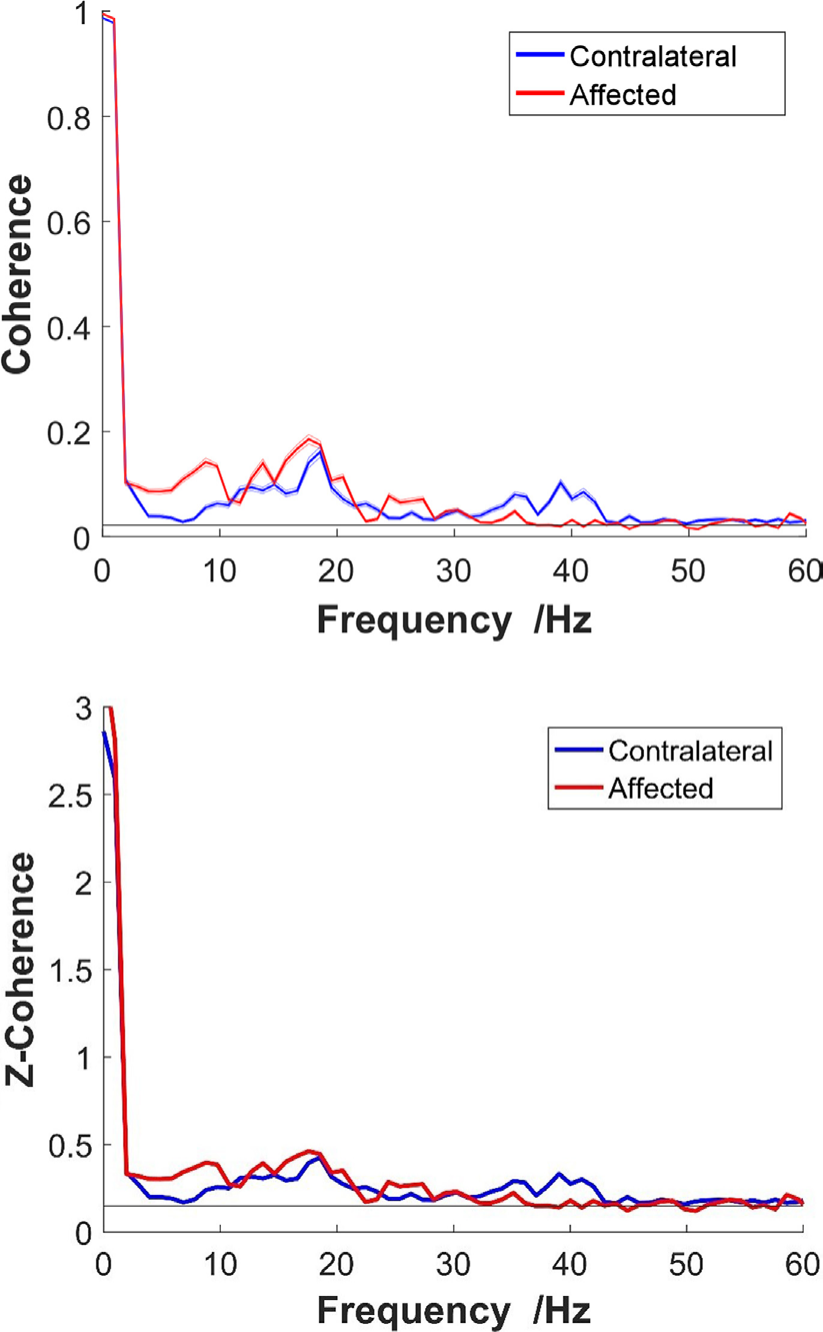
**Top: non-standardized coherence; bottom: z-coherence**. An exemplar frequency-coherence plots from a representative stroke participant at 40% maximum voluntary contraction. The solid thick red line represents the coherence of the affected side, and the blue line represents the contralateral side. Thin lines present SE.

The magnitude of coherence increased substantially with the number of MU spike trains selected. Therefore, using more MUs for coherence calculation was recommended (29, 30), providing a better estimation than a small number of MUs. Moreover, the coherence values can only be compared across different trials when the same amount of MU spike trains was analyzed. Since some trials only had a small number of accepted MU spike trains, eight MUs were selected for coherence calculation. For those trials with more than eight accepted MUs, the same amount of MU spike trains was randomly chosen from accepted MUs pool. A total of eight spike trains were randomly separated into two groups and then summed up into two composite spike trains (CSTs). The Welch’s averaged, modified periodogram method (31), adopted by multiple previous MU coherence studies (16, 17, 22), was performed to calculate the magnitude of squared coherence *C_*xy*_(f)* between the two CSTs:

(1) $$\begin{array}{l} C_{xy}(f)=\frac{{\left|P_{xy}(f)\right|}^{2}}{P_{xx}(f)P_{yy}(f)}, \end{array}$$

where *P_*xy*_(f)* is the cross-spectrum mean of two CSTs, and *P_*xx*_(f)* and *P_*yy*_(f)* are, respectively, their autospectrum densities. The calculation of the coherence-square used the MATLAB function “mscohere” with a length of 1,024 sample segments tapered by a Hann window and overlapped by 75% to estimate the entire frequency spectrum. The data processing steps are summarized in Figure 2. To obtain a better coherence estimate, the parameter selection for the coherence calculation was based on a previous study (32). In addition, to reduce the effect caused by random selection, 100 repeated tests were operated and averaged to acquire final coherence estimation for each trial. [An exemplar coherence estimation of the affected and contralateral sides of a stroke survivor is shown in Figure 3 (top)].

The confidence limit for coherence estimate was

(2) $$\gamma_{1-\alpha }^{2}=1-\alpha^{\left[1/\left(\text{EDOF}-1\right)\right]},$$

where α is (1 − α)% confidence level, and EDOF is the equivalent degree of freedom for Hann window (33).

Four different bands—delta band (1–4 Hz), alpha band (8–12 Hz), beta band (15–30 Hz), and gamma band (30–60 Hz)—were analyzed separately due to their different physiological meanings. We began by exploring the potential influence of three factors (two sides, four bands, and three MVC levels) on coherence. The magnitude of coherence for each band was quantized by its mean band coherence:

(3) $$\text{MBC}=\frac{\int_{f_{1}}^{f_{2}}C_{xy}\left(f\right)df}{B},$$

where *C_*xy*_(f)* is the magnitude of squared coherence, *B* is the width of one frequency band, and *f*_1_ and *f*_2_ are the lower and upper bounds of the corresponding band, respectively.

#### Correlation Analysis

A linear regression was performed to investigate the relationship between the possible factors (age, years poststroke, and the severity of impairment measured with Fugl-Meyer assessment) and the increased percentage of coherence on the paretic side compared with the contralateral side. We chose Fugl-Meyer assessment instead of Chedoke because the values of Fugl-Meyer assessment spread across a wider range from 1 to 66, which can give a better resolution for linear regression, and the correlation coefficient between Fugl-Meyer and Chedoke was 0.82. Therefore, only Fugl-Meyer was included in the regression.

#### Statistics

The coherence estimate was examined mainly on three aspects: comparisons of coherence amplitude across different MVC levels, comparisons between the affected and contralateral side, and comparisons of coherence amplitude across different frequency bands. The differences were tested statistically using a three-way repeated measures ANOVA, with *post hoc* pairwise comparisons conducted using Bonferroni correction method. A significance level of *p* = 0.05 was used. To satisfy the ANOVA test assumption, all coherence values (*C*) were transformed to Fisher’s values (FZ), which has been used in previous MU coherence studies (16, 34, 35). The fisher’s z-transformation equation is

(4) $$\text{FZ}=\text{arctan}h\sqrt{C}.$$

## Results

After decomposition and cross validation between STA and Delsys, the overall number of accepted MU spike trains is shown in Table 2. Low contraction levels that yielded a small number of MUs caused two main issues: (1) the number of accepted MUs cannot satisfy the requirement of coherence calculation (≥8). The “<8” column in Table 2 presents the number of trials with number of MUs < 8; (2) the random grouping algorithm for coherence calculation may be biased due to a small MU pool. Therefore, 40, 50, and 60% MVC were used for the final data analysis, while 20 and 30% MVC were excluded. (See Section “Discussion” for further details regarding the choice of MU numbers.) For these participants, under 40, 50, and 50% contraction levels, the average number of accepted MU spike trains was 17.73 ± 5.57 per single trial for the affected side and 20.27 ± 6.38 for the contralateral side; while the corresponding mean firing rates were 13.55 ± 4.04 pulse per second (pps) and 12.90 ± 3.31 pps, respectively.

**Table 2:** **The number of accepted motor units across different maximum voluntary contraction levels on two sides**.

	Affected side	Contralateral side	<8
20%	15.24 ± 4.51	18.71 ± 6.32	4
30%	16.76 ± 3.33	19.24 ± 6.10	3
40%	17.40 ± 4.94	20.10 ± 5.80	0
50%	17.76 ± 5.45	20.22 ± 6.33	0
60%	18.02 ± 6.26	20.50 ± 7.07	0

A three-way repeated measures ANOVA was tested across three isometric contraction levels (40, 50, and 60%) on two sides and four different frequency bands. The ANOVA results showed that there is an interaction [*F*(3,39) = 9.708, *p* < 10^−4^] between the side and frequency band. However, the third factor MVC showed no significant difference [*F*(2,26) = 1.666, *p* = 0.209], and also showed no interaction with other factors {[*F*(2,26) = 0.030, *p* = 0.971] between MVC and side; [*F*(6,78) = 0.982, *p* = 0.443] between MVC and frequency band; and [*F*(6,78) = 0.086, *p* = 0.997] for all three}.

**Figure 4:**
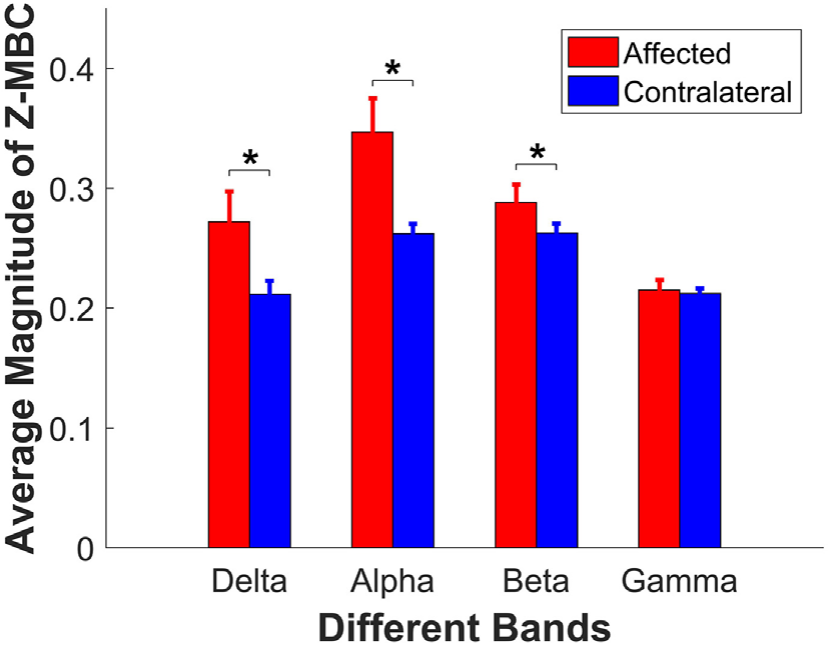
**Comparison of the z-coherence of different frequency bands between affected side and contralateral sides**. Each bar presents its corresponding average magnitude of mean band z-coherence (z-MBC), and the error bar presents its SE across 14 participants. Asterisks indicate the significant difference.

The interaction (side × frequency band) led us to conduct further *post hoc* pairwise comparisons separately. First, we compared the difference between paretic side and contralateral side for each individual band. The pairwise comparisons showed that the coherences were significantly different for delta band (*p* = 0.013), alpha band (*p* = 0.005), and beta band (*p* = 0.022), between the affected and contralateral sides, but not different for gamma band (*p* = 0.717). The average z-transformed mean coherence across all participants for delta band, alpha band, and beta band on affected side were 0.2720 ± 0.0254, 0.3468 ± 0.0282, and 0.2881 ± 0.0150, which were higher than those on the contralateral side (0.2116 ± 0.0112, 0.2620 ± 0.0082, and 0.2625 ± 0.0078, respectively, as shown in Figure 4). The baseline coherence was 0.2123 ± 0.004 and is comparable to the gamma band coherence.

**Figure 5:**
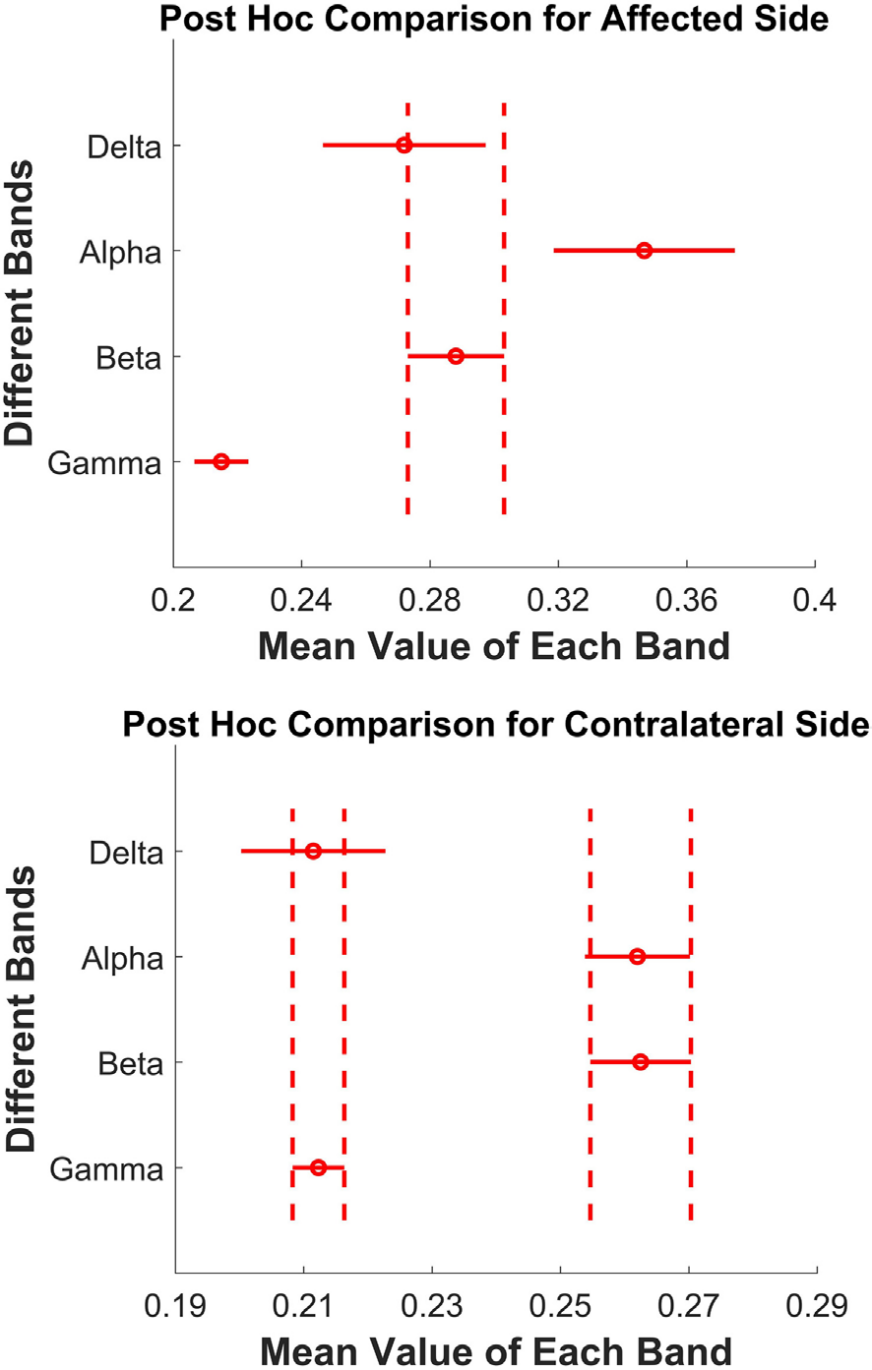
**Top: paretic side; bottom: contralateral side**. *Post hoc* pairwise comparison across four bands. Each red circle presents the mean value of one frequency band, and the red horizontal line is its corresponding 95% confidence interval. The vertical dashed lines show the overlap of two bands, which means no significant difference.

Second, *post hoc* pairwise comparisons were tested across four bands on two sides separately, and both showed a significant difference. For the paretic side, Figure 5 (top) shows *post hoc* evaluation, which revealed that (1) the alpha band had the highest mean coherence (*p* < 0.05); (2) the gamma band had the lowest mean value (*p* < 0.05); (3) the magnitude of delta band and beta hand had no statistical difference (*p* = 0.284). For the contralateral side, the *post hoc* comparison (Figure 5, bottom) showed that (1) the alpha and beta bands had significantly high coherence compared with the delta band and gamma band (*p* < 0.001); (2) the magnitude of the alpha and beta bands, or the delta and gamma bands had no statistical difference (*p* > 0.9).

Third, a linear regression was performed between three possible factors (age, years of poststroke, and the severity of stroke measured using Fugl-Meyer) and the increased percentage of coherence on paretic side compared with contralateral side. Separate regression was performed on the four different bands. Table 3 shows the fitting coefficients with the *r*^2^ value of each frequency band. The *r*^2^ values of the linear regression for the delta, alpha, beta, and gamma bands were 0.372, 0.364, 0.264, and 0.505, respectively. The results showed that the three factors tended to have a linear relation with the increased coherence value.

**Table 3:** **The fitting coefficients with corresponding *p* values, the *r*^2^ value of the linear regression on four bands**.

	Age	Years since stroke	Fugl-Meyer	*r*^2^
Delta	−0.0035 (0.0755)	0.0175 (0.3632)	0.0057 (0.2854)	0.372
Alpha	−0.0202 (0.2460)	0.0064 (0.7150)	0.0083 (0.1064)	0.364
Beta	−0.0133 (0.1278)	0.0074 (0.3969)	0.0014 (0.5470)	0.264
Gamma	−0.0120 (0.0314)*	0.0081 (0.1407)	0.0022 (0.1507)	0.505

*Individual p-values are shown in parentheses*.

*Significance at 95% level.

## Discussion

The coherence of MU spike trains is considered to partly reveal the relations between the motor neuron pool and spinal/superaspinal network, and the coherence of each frequency bandwidth can reflect the relative connectivity between the motor neuron pool and the upstream circuitry. Our current study quantified the changes of the coherence of each frequency band on the affected side, compared with the contralateral side of hemiparetic stroke survivors. Our results provided strong evidence that the coherences of three frequency bands (delta, alpha, and beta) showed a significant increase compared with the contralateral side. The coherence of the delta band and the alpha band increased substantially on the affected side (increased by 28.5 and 32.4%, respectively), compared with the contralateral side. The coherence in the beta band on the affected side also revealed moderate increase (by 9.6%), compared with the contralateral side. However, the magnitude of the gamma band coherence remained unchanged. Our general findings indicate that there are substantial changes in the common input to the motor neuron pool, arising from the spinal and supraspinal circuitry, which can modify the control of muscle activations after a stroke.

### Influence of Muscle Activation Level on Coherence

We found that the coherence estimates are consistent across a range of medium–high force levels (40–60%), indicating that the relative contribution of common synaptic input to the motor neuron pool is not sensitive to the levels of muscle contraction. The findings suggest that we can use a single prespecified muscle activation level (40–60% MVC) to estimate the coherence at different frequency ranges. It should be emphasized that the MVC levels affect the number of recruited MUs. During the coherence analysis, only a fixed number of MUs are randomly selected to the pool. Therefore, if the number of MUs used for the coherence analysis was identical, the MVC level within 40–60% MVC did not influence the estimate of coherence. However, the coherence estimate at force levels outside of the range was not examined, because lower force levels tend to yield a smaller number of MUs (see Table 2, some 20 and 30% trials cannot meet the minimum requirement), which may bias the coherence calculation. In contrast, higher forces tend to induce early muscle fatigue, which has been shown as a factor that can affect MU coherence (17). These factors can potentially bias our current coherent estimation, due to the sampling requirement of the coherence analysis and a relative steady MU discharge rate based on the assumption of the discharge spectrum analysis.

### Physiological Implications for the Variation of Each Band

Although the partition of frequency band range from different studies was not identical, the four specific ranges were divided similarly, and the corresponding physiological concepts have been widely accepted (17, 19, 22). In our present study, the gamma band always maintained at a stable and low power level on both paretic and contralateral sides. A substantial increase of the delta and alpha bands coherence was observed in the affected muscle in our stroke cohort. It is believed that the delta coherence is associated with the modulation of mean firing rate of the MUs. The increased delta coherence may reflect a compensatory change in response to the reduced firing rate and the reduced modulation of firing rate of MUs observed in stroke survivors (36). The large SE (see Figure 4) on the affected side also illustrated that the increased rate of these common inputs was different across participants, possibly due to the different severity of stroke. The alpha band coherence is associated with afferent feedback/spinal reflex contributions. In addition, previous studies indicated that the coherence between MUs increased with increasing muscle spindle activities (20, 22, 37). A flexed finger posture, potentially due to hyperreflexia, is a common feature in spastic stroke survivors. In addition, hyperreflexia is one common pathology in stroke survivors (38, 39), and it is conceivable that there is an increased spinal reflex contribution to the MU activation. However, we do not have direct evidence that the FDI muscle is hyperreflexia in our stroke cohort, since spasticity is not routinely assessed in the finger muscles.

The increase of common synaptic inputs affected beta band that revealed cortical and subcortical activities and short-term MU synchronization. The increased coherence in beta band results provided a consistent evidence with previous studies, which reported that the coherence of MU firing times had significant linear relationship with shared motor neuron inputs (6, 40). The stronger correlated higher level input to the FDI motor neuron pool may reflect more centralized control with a few cortical neurons directly projecting to the whole motor neuron pool, rather than having a complex network directly and indirectly modulating the activation of the motor neuron pool. In addition, a reduction of inhibitory high level input, due to stroke, could also contribute to more synchronized input. Clearly, additional studies are necessary to verify these potential changes.

### Correlations with Subject Demographic Information

To identify potential associations between the change of coherence in the affected FDI and the participant clinical information, separate multiple linear regression was performed for each coherence bandwidth. A moderate overall correlation was found as indicated by the *r*^2^ values. However, the individual demographic factors were not significant predictors on the change of coherence. A lack of strong correlations may arise from multiple aspects. First, the heterogeneous features of our stroke cohort of 14 participants may limit our correlation estimates, and a larger sample size may yield a better fit. Second, the change of coherence at different bandwidth may have stronger associations with other clinical features such as the lesion location or the integrity of the cortical spinal track, and the Fugl-Meyer or time poststroke may not be specific enough to show a strong correlation.

## Conclusion

In conclusion, we observed a significant increase of MU spike train coherence on the affected side of stroke survivors in the delta band, the alpha band, and the beta band, compared with the contralateral side. Our findings indicate that changes at multiple levels, including spinal and supraspinal levels, can all contribute to altered activation of affected muscles in stroke survivors. Further studies may be necessary to investigate possible relations between common drive and other frequency bands for stroke survivors and to explore if the increase in common drive on paretic side can lead to MU synchronization across different muscles.

## Author Contributions

CD analyzed the data and drafted the manuscript; NS, AS, and XH acquired the data; CD, NS, AS, WR, and XH discussed the study and revised the manuscript.

## Conflict of Interest Statement

The authors declare that the research was conducted in the absence of any commercial or financial relationships that could be construed as a potential conflict of interest.
